# Long‐term data reveal fitness costs of anthropogenic prey depletion for a subordinate competitor, the African wild dog (*Lycaon pictus*)

**DOI:** 10.1002/ece3.11402

**Published:** 2024-06-25

**Authors:** Johnathan Reyes de Merkle, Scott Creel, Matthew S. Becker, Ben Goodheart, Thandiwe Mweetwa, Henry Mwape, Egil Dröge, Twakundine Simpamba

**Affiliations:** ^1^ Zambian Carnivore Programme Mfuwe Eastern Province Zambia; ^2^ Department of Ecology Montana State University Bozeman Montana USA; ^3^ Institutionen för Vilt, Fisk Och Miljö, Sveriges Lantbruksuniversitet Umeå Sweden; ^4^ Wildlife Conservation Research Unit Oxford University Oxford UK; ^5^ Department of National Parks and Wildlife, South Luangwa Area Management Unit Mfuwe Eastern Province Zambia

**Keywords:** competition, conservation, population demography, predator ecology, prey depletion, protected areas

## Abstract

Within carnivore guilds, dominant competitors (e.g., lions, *Panthera leo*) are limited primarily by the density of prey, while subordinate competitors (e.g., African wild dogs, *Lycaon pictus*) have been limited by the density of dominant competitors. Historically, the fitness and population density of subordinate competitors have not been tightly linked to prey density. However, populations of large herbivores have declined substantially across sub‐Saharan Africa due to human impacts, and where prey depletion is severe, fitness costs for competitive subordinates may begin to outweigh the benefits of competitive release. Using long‐term intensive monitoring of African wild dogs in Zambia's Luangwa Valley Ecosystem (LVE), we tested the effects of prey depletion on survival and reproduction. We hypothesized that African wild dog fitness would be lower in prey‐depleted areas, despite lower lion densities. Our study area included four contiguous regions that varied in protection level, prey density, and lion density. We fit Bayesian Cormack–Jolly–Seber and closed‐capture models to estimate effects on survival and population density, and generalized linear models to estimate effects on reproductive success. We found that the LVE is a stronghold for African wild dogs, with an estimated median density of 4.0 individuals/100 km^2^. Despite this high density, survival and reproduction differed among regions, and both components of fitness were substantially reduced in the region with the lowest prey density. Anthropogenic prey depletion is becoming an important limiting factor for African wild dogs. If prey depletion (or any other form of habitat degradation) becomes severe enough that its fitness costs outweigh the benefits of competitive release, such changes can fundamentally alter the balance between limiting factors for competitively subordinate species.

## Graphical abstract

In the past, the fitness of subordinate competitors in the African large carnivore guild has been more strongly related to the density of dominant competitors than to the density of prey. Using long‐term data, we compared the survival and reproduction of wild dogs across four regions of varying protection status, prey density, and lion density. Wild dog survival and reproduction were both reduced in areas affected by anthropogenic prey depletion; revealing that prey depletion can become severe enough that its fitness costs outweigh the benefits of competitive release, such changes can fundamentally alter the balance between limiting factors for competitively subordinate species.

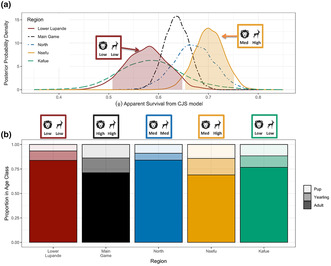

## 1 INTRODUCTION

Biodiversity loss is a global, human‐induced crisis characterized by accelerated rates of population decline and extinction across a wide range of species (Ceballos et al., 2017; Rosenberg et al., 2019; Singh, 2002). Large carnivores face a particularly high risk of extinction due to their small populations, large area requirements, and dependence on relatively intact ecosystems that support sufficient prey (Purvis et al., 2000). Consequently, these species have experienced drastic range reductions and population declines driven by habitat loss and fragmentation, overexploitation, persecution, and prey depletion (Ripple et al., 2014; Wolf & Ripple, 2017). Prey depletion itself is driven by a combination of habitat loss and overexploitation. The illegal bushmeat trade has altered ecosystem stability and function by depleting the large herbivore guild through poaching (Bogoni et al., 2020; Effiom et al., 2013; Lindsey et al., 2011, 2013; Ripple et al., 2015, 2016; Wolf & Ripple, 2016), leaving “empty ecosystems” with the vegetation intact but lacking the herbivores that are essential for normal ecosystem function (Redford, 1992; Wilkie et al., 2011). Many ecosystems that are central to the conservation of African large carnivores have experienced substantial and accelerating prey depletion (Effiom et al., 2013; Lindsey et al., 2011, 2013; Ripple et al., 2014, 2015, 2016; Wolf & Ripple, 2016, 2017).

Apex carnivores are usually limited by prey density, creating a strong, positive correlation between predator and prey densities (Carbone et al., 2011; Carbone & Gittleman, 2002; Karanth, 1999). Cheetahs, African wild dogs, and dholes are notable exceptions to this rule because they are competitively subordinate within their guilds (Creel & Creel, 1996; Durant, 2000; Steinmetz et al., 2013). Top‐down forces due to intraguild predation and kleptoparasitism by larger carnivores (e.g., tigers, lions, and spotted hyenas) have strong effects on their population dynamics and densities (Hairston et al., 1960; Palomares & Caro, 1999; Polis et al., 1989), and (as it is typical of subordinate competitors) they use a combination of dietary, spatial, and temporal niche partitioning to coexist with dominant competitors (Bhandari et al., 2021; Broekhuis et al., 2013; Dröge et al., 2017; Fedriani et al., 2000; Goodheart et al., 2022; Hayward & Slotow, 2009; Karanth & Sunquist, 2000; Vanak et al., 2013). True apex carnivores like the lion typically exploit areas with high prey density (Carbone et al., 2011; Carbone & Gittleman, 2002; Karanth, 1999), so competitive subordinates must optimize a trade‐off between avoiding dominant competitors and maintaining access to prey (Bhandari et al., 2021; Creel & Creel, 1996; Durant, 2000; Laurenson, 1995; Swanson et al., 2014).

The African wild dog (*Lycaon pictus*) is an endangered social canid consistently found at low population densities, with behavior and ecology that are strongly shaped by competition with larger competitors (especially lions) (Creel & Creel, 2002). Within ecosystems, African wild dogs often select areas with low lion density, and consequently low prey density (Creel & Creel, 1996). Across ecosystems, African wild dog densities are highest in areas with low densities of lions and spotted hyenas (Creel & Creel, 1996). The strong, consistent, and positive correlation of lion and hyena density with prey density makes it clear that these species are likely to be limited by prey depletion (Carbone et al., 2011; Carbone & Gittleman, 2002; Ferreira & Funston, 2010; Orsdol et al., 1985). In contrast, the African wild dog has long been considered able to persist in areas of low prey density due to the benefits of competitive release (Creel & Creel, 2002; Marneweck et al., 2022; Swanson et al., 2014). Recently, as prey depletion due to heavy illegal offtake has become stronger, limiting effects on African wild dog density and dynamics have begun to emerge (Goodheart et al., 2021). Under the ecological conditions of the past, there was strong evidence that the benefits of competitive release outweighed the costs of resource limitation for African wild dogs (Creel & Creel, 1996, 2002): if this balance of costs and benefits is now being reversed by prey depletion, recognition of this change will be essential for their conservation (Creel et al., 2023a, 2023b; Goodheart et al., 2021). Range‐wide, African wild dogs now occupy areas that span a gradient of protection, all increasingly exposed to the effects of anthropogenic prey depletion. Measuring region‐specific demographic rates for African wild dogs across levels of anthropogenic prey depletion can inform assessments of the effectiveness of conservation efforts (Pulliam, 1988).

Protected areas (PAs) are the bedrock of conservation, but populations within PAs are increasingly isolated and affected by anthropogenic processes, as adjacent areas experience habitat loss and conversion due to expanding human populations and consequent edge effects (Cardillo et al., 2004; Craigie et al., 2010; Jones et al., 2018; Newmark, 2008; Powers & Jetz, 2019; Watson et al., 2013, 2015; Western et al., 2009; Williams et al., 2017; Wittemyer et al., 2008). The International Union for the Conservation of Nature (IUCN) classifies PAs based on the level of protection, management, and conservation goals (Dudley, 2008), with categories I to III considered “high‐protection” and categories IV to VI considered “low‐protection.” Although these categories do not guarantee that the nominal level of protection is actually provided (Dudley et al., 2010; Watson et al., 2014), PAs are associated with higher biodiversity than unprotected areas (Gray et al., 2016), in a manner that is affected by funding, size, management, and insulation from human activities (Joppa & Pfaff, 2009; Leberger et al., 2020; Visconti et al., 2019). Despite its essential role in conservation, the world's current PA network does not protect 40% to 56% of mammals from the threat of anthropogenic extinction (Williams et al., 2022).

In Zambia, PAs form a network with areas of high protection (i.e., National Parks ~IUCN category II) surrounded by areas of low protection (i.e., Game Management Areas ~IUCN category VI) that serve as corridors and buffer zones. Game Management Areas (GMAs) have much more habitat alteration and human activity than National Parks, particularly wire‐snare poaching (Watson et al., 2013, 2015). Prior studies have linked increased poaching pressure in and around Zambian PAs to declines in both large herbivores and large carnivores (Becker et al., 2013; Creel et al., 2018; Goodheart et al., 2021; Rosenblatt et al., 2016, 2019; Vinks et al., 2020, 2021), and in the Luangwa Valley of eastern Zambia, GMAs have been shown to support low large herbivore densities due to heavy wire‐snare poaching (Rosenblatt et al., 2019; Watson et al., 2013). The Luangwa Valley Ecosystem (LVE) has long been considered a stronghold for African wild dogs, but there has been no rigorous description of their density, demography, or ecology (Strampelli et al., 2022). The LVE African wild dog population occupies four relatively distinct regions (demarcated by natural and human‐made boundaries) that vary in protection level, prey density, and lion density. These four regions provide an opportunity to test whether anthropogenic prey depletion is becoming strong enough that its costs outweigh the benefits of competitive release. We hypothesize that prey depletion incurs strong fitness costs, even in areas of relatively low lion density. If the hypothesis is supported, then areas of low densities of dominant competitors and prey due to high levels of anthropogenic prey depletion will not be likely to support viable African wild dog populations. Therefore, the well‐established pattern that African wild dogs fare best in areas with low densities of dominant competitors will no longer provide unambiguous guidance for conservation strategy (Creel et al., 2023b; Goodheart et al., 2021, 2022).

Here, we use data from a large‐scale, long‐term study of individually recognized African wild dogs in the LVE to (1) provide the first rigorous estimates of population density in this continentally important population, (2) provide rigorous estimates of annual rates of survival and reproduction, (3) test for variation in fitness between regions that vary in the densities of prey and competitors, and (4) relate these results to prior research to test the hypothesis that current levels of prey depletion carry costs that outweigh the benefits of competitive release. Because interspecific competition is strong in many guilds, our results are likely to be pertinent to the conservation of many species that are limited by the balance between resources and dominant competitors, with increasing human effects on both.

## 2 RESULTS

### 2.1 Population density

**FIGURE 1:**
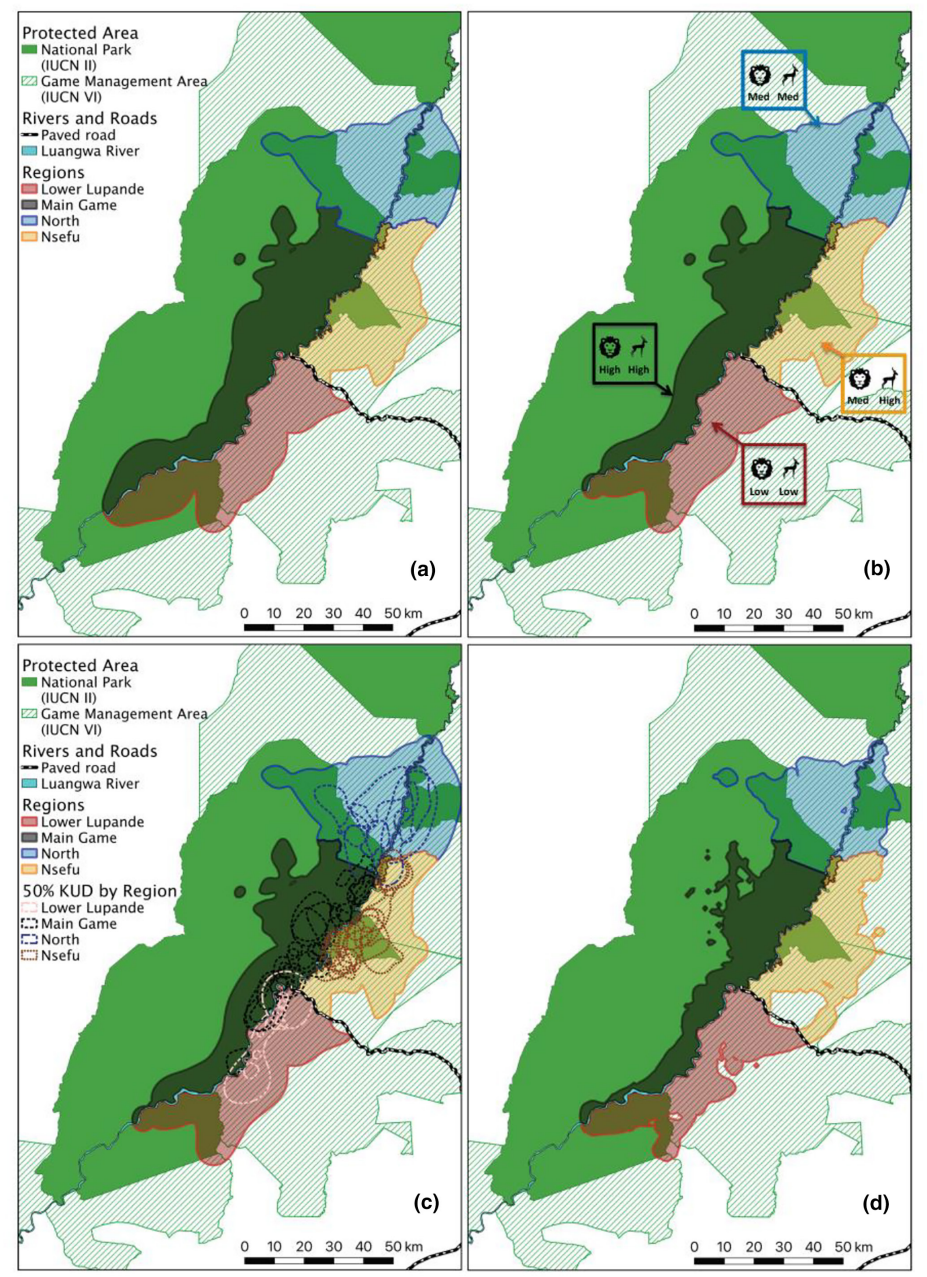
Maximum extent of the demographic monitoring area with regional boundaries in the Luangwa Valley Ecosystem (LVE). (a) Study area based on 95% isopleths of African wild dog packs' annual home ranges generated by kernel utilization distributions (KUDs), merged across 7 years (2014–2020). (b) Study area based on 95% isopleths of African wild dog packs' annual home ranges from KUDs, merged over 4 years (2016, 2018–2020). (c) The maximum extent of study area from the 4 years (2016, 2018–2020) regions as in B, overlaid with corresponding packs' 50% isopleths generated by KUDs, with colors coordinated to assigned regions. (d) Study area based on 95% isopleths of African wild dog packs' annual home ranges generated by dynamic Brownian bridge movement models (dBBMMs), merged over 4 years (2016, 2018–2020). Solid green shows National Parks (IUCN II) and hashed light green shows Game Management Areas (IUCN VI) overlapping the study area. The main Luangwa River is denoted in light blue, and the major paved road is in black and white. The four regions are shown by color: Lower Lupande = Red, Main Game = Black, North = Blue, and Nsefu = Orange.

For the LVE study area as a whole (Figure 1), African wild dog density was high. The median density ($\hat{D}$) was 2.98 adults and yearlings/100 km^2^ (95% CrI: 2.67–3.85; *N* = 7 years, 2014–2020) when estimating the area occupied with KUDs (Table 1). The median African wild dog density was 4.01 adults and yearlings/100 km^2^ (95% CrI: 3.50–5.43; *N* = 4 years) when estimating the area occupied with dBBMMs, which could be fit to data for only 4 years (Table 1). For the same 4 years, median density based on KUDs (2.78 adults and yearlings/100 km^2^, 95% CrI: 2.45–3.71) was very similar to the estimate for the entire 7 years (Table 1).

**TABLE 1:** African wild dog density (adults and yearlings/100 km^2^) in the Luangwa Valley Ecosystem.

Period (years)	Method	Median	Mean (95% credible interval)
7	KUD	2.98	3.05 (2.67–3.85)
4	KUD	2.78	2.86 (2.45–3.71)
4	dBBMM	4.01	4.12 (3.50–5.43)

*Note*: Density is averaged over 7 years (2014–2020) or over 4 years (2016, 2018–2020). Method refers to techniques used to create annual group home ranges; either kernel utilization distribution (KUD) or dynamic Brownian bridge movement model (dBBMM).

The median annual abundance ($\hat{N}$) estimated by capture mark recapture was 106 adults and yearlings (95% CrI: 96–136, *N* = 7 years) across all 7 years and 110 adults and yearlings (95% CrI: 99–144, *N* = 4 years) across the subset of 4 years for which the monitoring area could be estimated using dBBMMs. The mean annual demographic monitoring area across 7 years based on KUDs was 3675 km^2^. The maximum extent of the four regions that comprise the study area (Lower Lupande, Main Game, North, and Nsefu) is shown in Figure 1 for both the 7‐ and 4‐year periods (Figure 1a,b). The maximum extent of the demographic monitoring area was created by merging home ranges across all respective years (either 7 or 4 years). The core (50% isopleth) of each pack's range is superimposed in Figure 1c, showing that each pack resided largely within a single region. The extent of each of the four regions based on dBBMMs is shown in Figure 1d. As expected, estimates of the area occupied decreased when dBBMMs were fit to the same data used to fit KUDs (compare Figure 1b,d), and the mean area across 4 years (2016, 2018–2020) based on dBBMMs was 3076 km^2^.

### 2.2 Survival

#### 2.2.1 Age‐ and sex‐specific annual apparent survival (ϕ)

**FIGURE 2:**
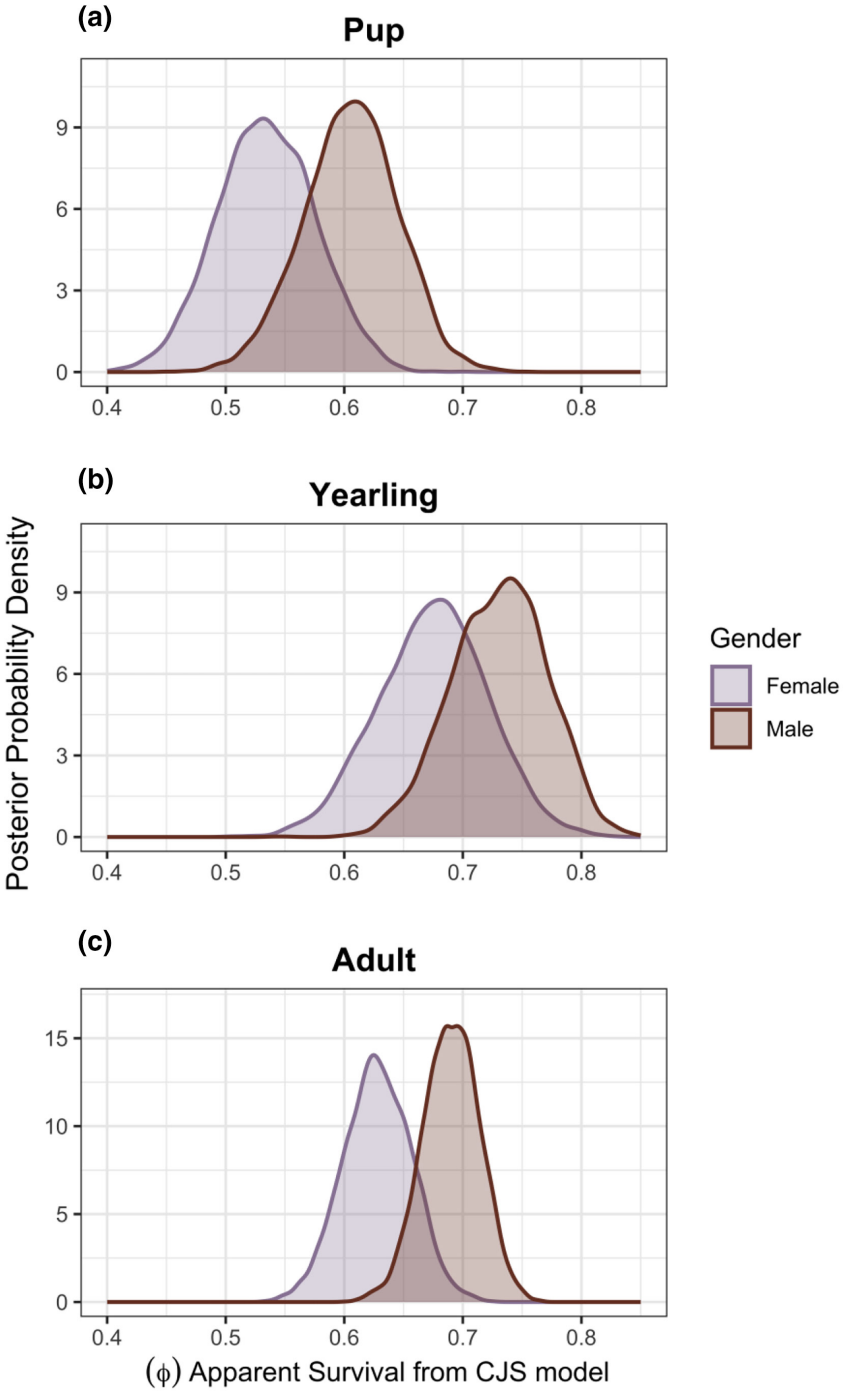
Posterior probability distributions of age‐ and sex‐specific annual apparent survival rates (ϕ) from a Cormack–Jolly–Seber model with individual random effects on detection probability (*p*). (a) Pups (<1 year old). (b) Yearlings (1–1.99 years old). (c) Adults (≥2 years old).

For the LVE study area as a whole, annual apparent survival rates (ϕ) estimated by capture–mark–recapture (CMR) were similar to those reported from other large and stable populations of African wild dogs, and variation in survival among ages and sexes followed the patterns reported by prior studies (Creel et al., 2004; McNutt & Silk, 2008; Woodroffe, 2011). Annual apparent survival rates were lowest for pups with a median of 0.570 (95% CrI 0.466–0.669), highest for yearlings with a median of 0.704 (95% CrI 0.597–0.796), and slightly lower for adults with a median of 0.662 (95% CrI 0.580–0.728) (Figure 2). Males (with a median of 0.684 [95% CrI 0.547–0.788]) had slightly higher annual apparent survival than females (with a median of 0.620 [95% CrI 0.475–0.740]) (Figure 2). The estimated median apparent annual survival (ϕ) for female pups (<1 year old) was 0.534 (95% CrI 0.455–0.615), and for male pups was 0.607 (95% CrI 0.524–0.680) (Figure 2a). The estimated median apparent survival (ϕ) for yearling (1–1.99 years old) females was 0.676 (95% CrI 0.584–0.763) and for yearling males was 0.732 (95% CrI 0.648–0.805), (Figure 2b). The estimated median apparent survival (ϕ) for adult (≥2 years old) females was 0.628 (95% CrI 0.572–0.683) and for adult males was 0.690 (95% CrI 0.642–0.736) (Figure 2c). The mean monthly detection probability (*p*) was .539 (95% CrI 0.497–0.580), and when annualized, the mean detection probability was .999 (95% CrI 0.998–1), indicating that monitoring was sufficiently intensive to detect virtually all resident African wild dogs within the monitoring area, after excluding peripheral areas with poor monitoring from this analysis. As in prior studies, the annual survival rate of radiocollared adults (0.75, 95% CrI 0.62–0.85) tended to be higher than the survival of uncollared adults (0.66, 95% CrI 0.61–0.70) (Creel et al., 1997; Goodheart et al., 2022; Woodroffe, 2001).

#### 2.2.2 Region‐specific annual apparent survival (ϕ)

**FIGURE 3:**
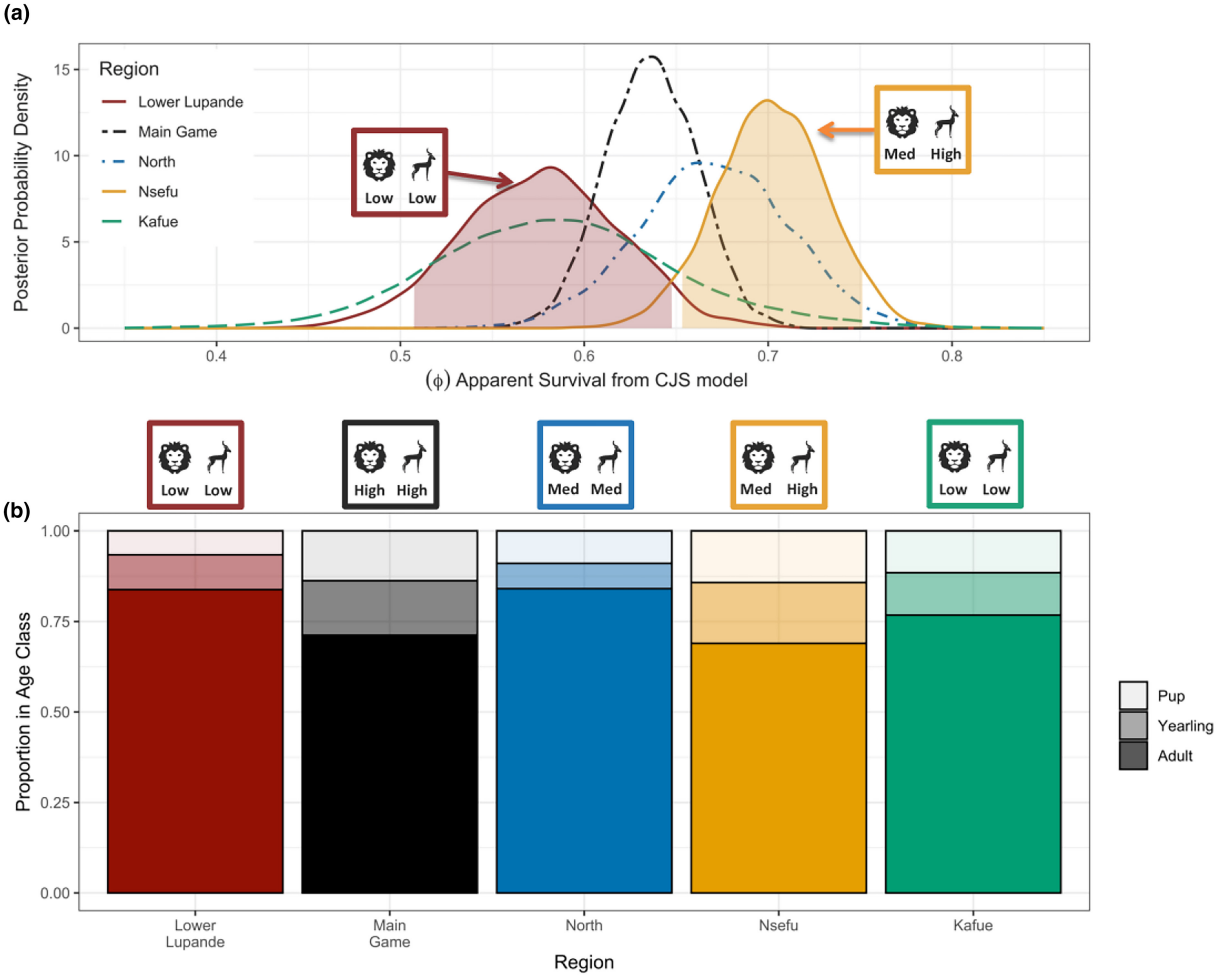
Area‐specific annual apparent survival (ϕ) and age‐class distributions. (a) Posterior probability distributions for annual apparent survival (ϕ) for the four regions of LVE (Lower Lupande = red, Main Game = Black, North = Blue, Nsefu = Orange) and the Greater Kafue Ecosystem (Green). The red and orange shading show the 90% credible intervals for the Lower Lupande and Nsefu regions. (b) Age class distributions for each of the four LVE regions and Kafue. Adults (≥2 years old) are at the bottom of each bar, yearlings (1 to 1.99 years old) in the middle, and pups (<1 year old) at the top. The Greater Kafue Ecosystem's annual apparent survival estimates and age class distributions are taken from Goodheart et al., 2021 (Goodheart et al., 2021).

Annual survival rates varied substantially among the four regions (Figure 3a). Survival was lowest in Lower Lupande (where prey density was lowest: see Section 4), highest in Nsefu (where prey density was highest), and intermediate in the Main Game and North regions (where prey density was intermediate). Estimated median annual apparent survival (ϕ) was 0.577 (95% CrI 0.491–0.657) in the Lower Lupande region, 0.636 (95% CrI 0.589–0.683) in the Main Game region, 0.670 (95% CrI 0.589–0.748) in the North region, and 0.702 (95% CrI 0.642–0.759) in the Nsefu region. There was no overlap between the 90% credible intervals for survival rates in Lower Lupande (0.504–0.645) and Nsefu (0.652–0.750).

The mean monthly detection probability (*p*) was very similar for this analysis and the previous model that estimated the effects of age and sex on survival was at 0.533 (95% CrI 0.489–0.577), and when annualized, the mean detection probability was 0.999 (95% CrI 0.998–1).

The Lower Lupande region consisted of 6.6% pups, 9.6% yearlings, and 83.8% adults. The Main Game region consisted of 13.7% pups, 15.1% yearlings, and 71.2% adults. The North region consisted of 8.9% pups, 7.0% yearlings, and 84.1% adults. The Nsefu region consisted of 14.2% pups, 16.8% yearlings, and 69.0% adults. All the regions had comparable sex ratios (43.9%, 45.3%, and 40.0% females for Lower Lupande, Main Game, and the North regions, respectively), with Nsefu region being the most female dominated (59.8%).

### 2.3 Reproduction

**FIGURE 4:**
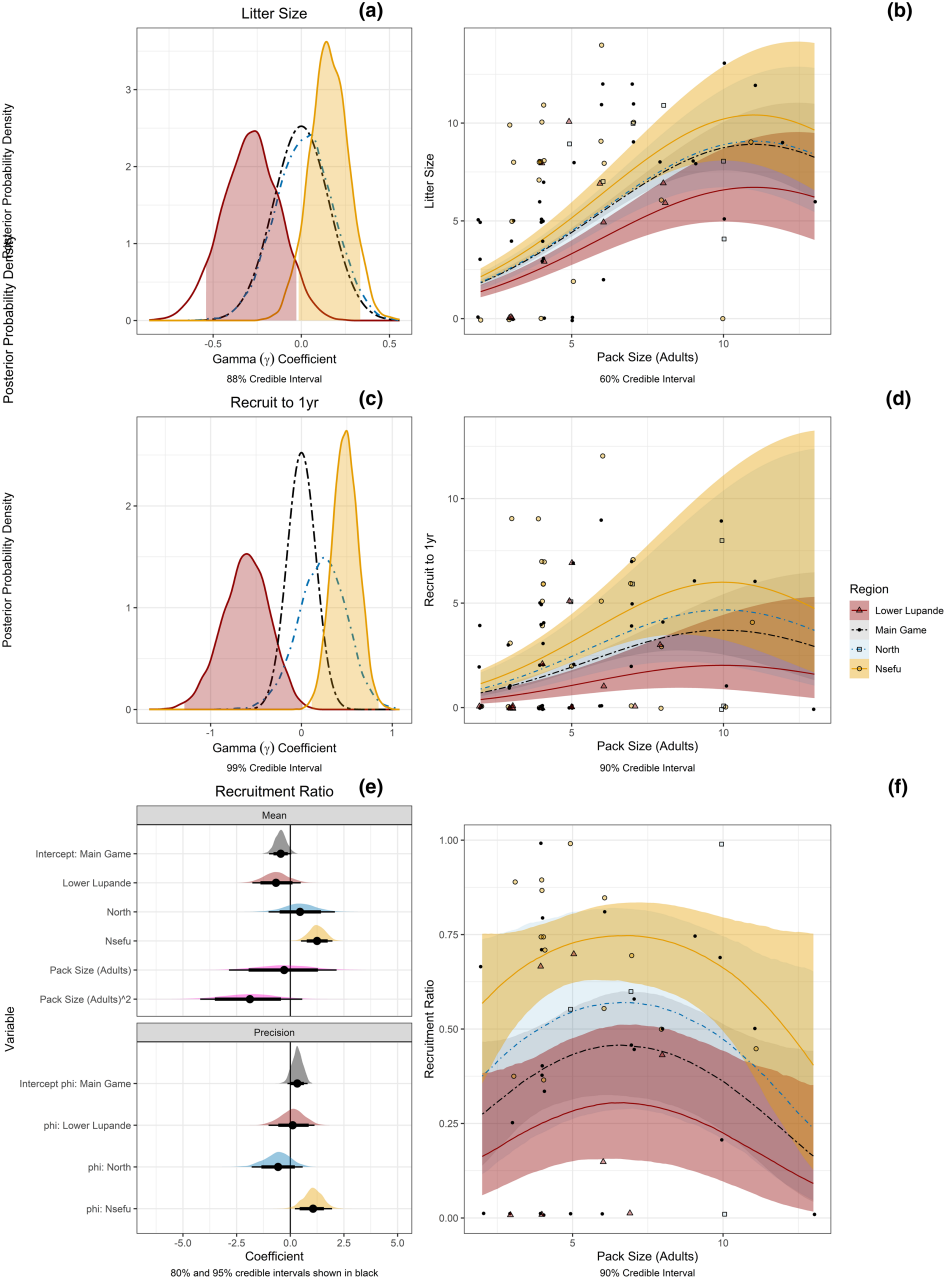
Differences between LVE regions in litter size, the number of pups recruited to 1 year, and recruitment ratio (proportion of pups raised to 1 year). For all panels, Lower Lupande = red, Main Game = black, North = blue, and Nsefu = orange. Panels b, d, and f show the raw data (jittered along x‐axis) together with the fitted GLM, and panels a, c, and e show unback transformed coefficients for each GLM. (a) Unback transformed coefficients for the effect of region on litter size in a Poisson GLM. Shading shows 88% credible intervals. (b) The Poisson GLM of litter size with effects of pack size (adults) and region. Shading shows 60% credible intervals for each region. (c) Unback transformed coefficients for the effect of region on the number of pups recruited to 1 year in a Poisson GLM. Shading shows 99% credible intervals. (d) The Poisson GLM of pups recruited to 1 year with effects of pack size (adults) and region. Shading shows 90% credible intervals. (e) Unback‐transformed coefficients for the two parameters (mean, μ and precision, φ) of a Beta GLM testing the effect of region on recruitment ratio, with coefficients for the linear and quadratic effects of adult pack size shown in pink. Thick and thin horizontal lines show 80% and 95% credible intervals for each coefficient. (f) The beta GLM of recruitment ratio with effects of region and pack size (adults). Shading shows 90% credible intervals.

Because pack size is known to have a strong effect on the number of pups born and raised, we included pack size in models that tested for differences between regions. The mean pack size was 5.27 adults and 2.33 yearlings for a total of 7.60 (range 2–23, *N* = 91 pack‐years) between 2008 and 2021. As with variation in survival rates, all measures of reproductive success (litter size at first count, number of pups raised to year, and the recruitment ratio) were best in Nsefu, worst in Lower Lupande, and intermediate in the other regions (Figure 4). For litter size at first count, the 88% credible intervals for Nsefu and Lower Lupande did not overlap for the effect of region (Figure 4a). When back transformed to estimate litter size dependent on the number of adults in a pack and region, the 60% credible intervals for Nsefu and Lower Lupande did not overlap for pack sizes with fewer than nine adults, a range that includes all the Lower Lupande data (Figure 4b). For pups recruited for 1 year, the 99% credible intervals for Nsefu and Lower Lupande did not overlap with the regional coefficient estimates (Figure 4c). When back transformed to estimate the pup recruitment to 1 year dependent on adult pack size and region, the 90% credible intervals for Nsefu and Lower Lupande did not overlap across pack sizes from three to eight adults, a range that included 92% of the Lower Lupande data (Figure 4d). For the recruitment ratio, the 95% credible intervals for Nsefu and Lower Lupande did not overlap with the regional coefficient estimates (Figure 4e). When back transformed to estimate the recruitment ratio dependent on adult pack size and region, the 90% credible intervals for Nsefu and Lower Lupande did not overlap across pack sizes from three to 10 adults, a range that included all the Lower Lupande data (Figure 4f). For each of these measures, the Main Game and North Regions were intermediate, with considerable overlap with both Nsefu, where reproduction was best, and Lower Lupande, where reproduction was worst. Linear and quadratic effects of pack size also affected each of these measures, and differences between regions in reproduction were most pronounced at intermediate pack sizes (3–8 adults), which were most common.

## 3 DISCUSSION

The high density of African wild dogs in the Luangwa Valley Ecosystem (LVE) makes it an important stronghold for the species. The estimated density of 4.0 adults and yearlings/100 km^2^ (based on dBBMMs) is the highest recorded for the species. Even the more conservative estimate of 3.0 adults and yearlings/100 km^2^ (based on KUDs) is among the highest densities recorded (Selous 3.8; Moremi 3.5; Samburu‐Laikipia 3.3 adult and yearlings/100 km^2^) (Creel et al., 2004; Goodheart et al., 2021; Woodroffe, 2011). The LVE also plays an important role in connectivity, as it is central to multiple Transfrontier Conservation Areas (TFCAs) shared with Malawi, Zimbabwe, and Mozambique. We have observed collared African wild dogs dispersing from the Luangwa Valley Ecosystem into Mozambique and the Mid‐Zambezi Valley (traveling 2163 km and reaching a point 337 km from their natal range), confirming that there is still functional connectivity between LVE and distant parts of these TFCAs (Creel et al., 2019).

The pattern in survival across ages and between sexes was similar to that reported for other high‐density populations (e.g., Selous: pups mean annual survival = 0.75 (95% CI 0.66–0.84); yearlings = 0.84 (95% CI 0.73–0.91), and adults = 0.71; and Moremi: pups = 0.48 (95% CI 0.42–0.54), yearlings = 0.74 (95% CI 0.72–0.79), and adults within a 95% CI of 0.40–0.67) (Creel et al., 2004; Creel & Creel, 2002; Goodheart et al., 2021). Males tended to have higher survival rates than females, although the difference was small. Yearlings had the highest survival rate, followed by adults, and pups had the lowest survival rate.

Within the LVE, a highly coherent pattern emerged from regional differences in survival and reproductive success. Estimates of survival and reproductive success (across three measures) were substantially lower in the region (Lower Lupande) with the lowest prey density, particularly when compared to the region (Nsefu) with the highest prey density. Despite the Lower Lupande region having the lowest lion density, the African wild dogs in the region had the lowest apparent survival. The median apparent survival rate in Lower Lupande was ~17.8% lower than that of Nsefu with no overlap of 90% credible intervals. We included the posterior probability distribution for annual survival of African wild dogs in the Greater Kafue Ecosystem (GKE) in Figure 3 to facilitate comparison to the four regions in LVE. The estimated median annual apparent survival (ϕ) in the GKE was 0.58 (95% CrI 0.46–0.73) for yearlings and adults. The estimated annual apparent survival (ϕ) of pups in GKE was 0.59 and within the range of yearlings and adults in the GKE. The age structure of the GKE African wild dog population consisted of 11.5% pups, 11.7% yearlings, and 76.8% adults.

These differences in survival rates between regions were not due to differences in age structure. The region with the highest survival rate (Nsefu) held the highest proportion of pups, and the region with the lowest survival rate held the lowest proportion of pups (Figure 3b). Because pups have appreciably lower survival than adults or yearlings, these age structures strongly suggest that rates of both survival and reproduction were highest in Nsefu, lowest in Lower Lupande, and intermediate in the other regions.

Apparent survival in Lower Lupande was comparable to that of African wild dogs in the GKE, which has experienced severe prey depletion and supports a very low density of African wild dogs even though it holds a low density of lions (Goodheart et al., 2021). In both Lower Lupande and the GKE, low lion densities are associated with anthropogenic prey depletion which also yields poor African wild dog survival. Our results support the hypothesis that the detrimental effects of very low prey density outweigh the benefits of competitive release.

**FIGURE 5:**
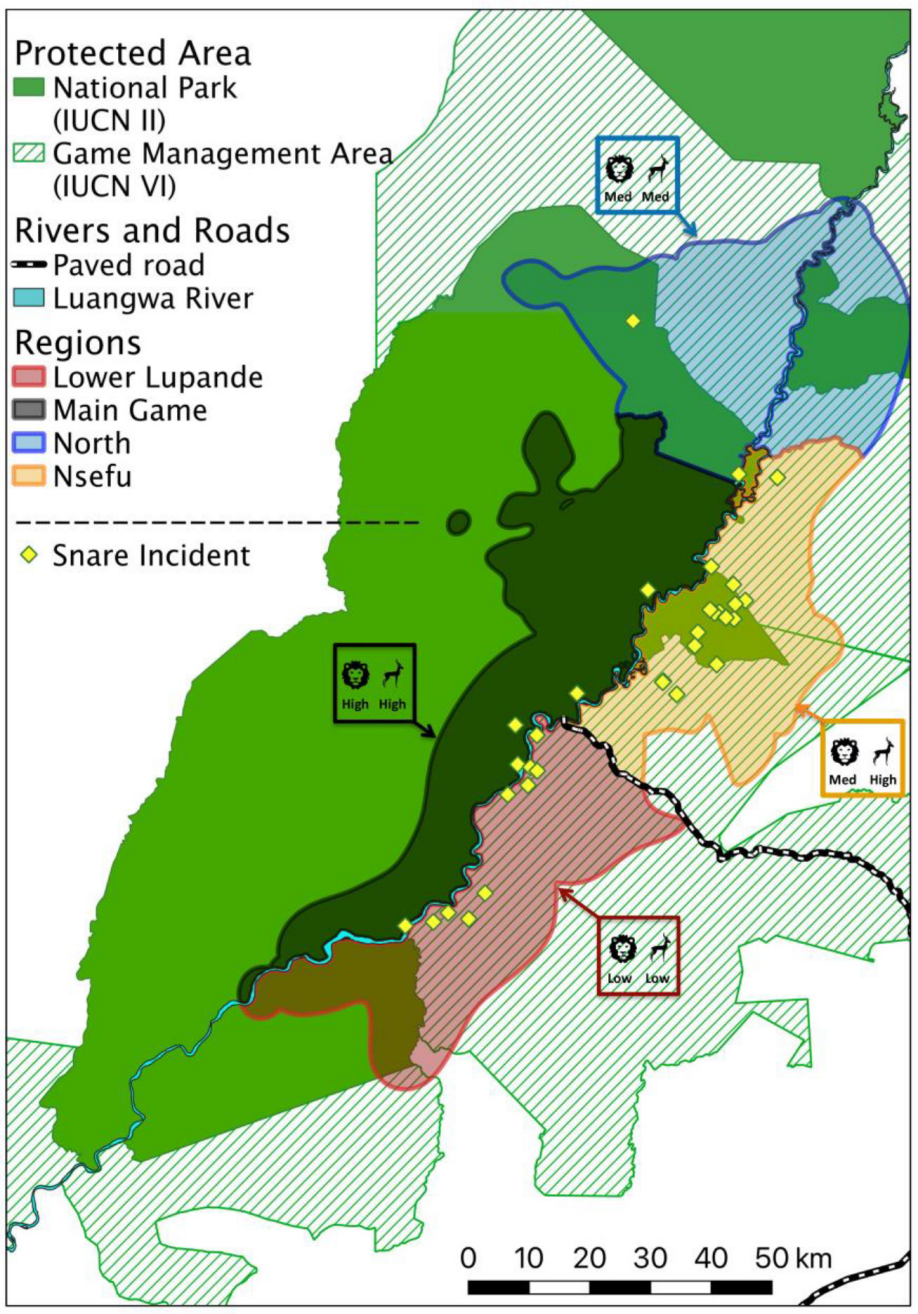
Known snare incidents for African wild dogs in LVE (2008–2020) overlaid with the four regions (Lower Lupande in red (low lion and prey density), Main Game in black (high lion and prey density), North in blue (intermediate lion and prey density), and Nsefu (intermediate lion density and high prey density) in orange shading) from the max extent KUD map of 7 years (2014–2020). Snare incidents are symbolized as a yellow diamond (including snare mortalities, injuries, snare removals, and individuals seen with an active snare). The Luangwa River is shown in light blue, and the main paved road is shown in black and white. The solid green represents national parks (IUCN II) and hashed light green for GMAs (IUCN VI).

In the Lower Lupande region, low prey density is driven primarily by illegal wire‐snare poaching, although habitat conversion also plays a role (Watson et al., 2013, 2015). To disentangle the effects of prey depletion versus direct killing or injury of African wild dogs by snares, we mapped the spatial distribution of African wild dog snare incidents. Snare incidents included snare mortalities, injuries, snare removals, and African wild dogs sighted carrying a snare wire. As shown in Figure 5, African wild dog snare incidents were quite evenly distributed between the Lower Lupande and Nsefu regions; and were very rare in the Main Game and North regions. Thus, variation among regions in prey density was a good predictor of survival, but variation in direct snaring was not. This result aligns with the prior finding that African wild dogs are directly snared at similar rates in the LVE (where their density is among the highest on record) and the Greater Kafue Ecosystem (where their density is among the lowest on record) (Creel et al., 2023b).

Like survival, reproductive success was lowest in the area with the lowest prey density, and highest in the area with the highest prey density. Lower Lupande had smaller litter sizes, fewer yearlings recruited, and lower recruitment ratios than Nsefu, after accounting for the effect of pack size, which is known to have a strong effect on African wild dogs' reproductive success (Courchamp & Macdonald, 2001; Creel et al., 2004; Creel & Creel, 2015; Gusset & Macdonald, 2010; Malcolm & Marten, 1982; McNutt & Silk, 2008).

Potential alternative causes of regional differences in survival and reproductive success include road mortality and disease (Creel & Creel, 1998; Fanshawe et al., 1991; Woodroffe, Davies‐Mostert, et al., 2007). Road mortality was very rare for African wild dogs in LVE, with only two recorded incidents (one in the Main Game and one in the Lower Lupande region). Most of the gravel and paved roads within the study area are used for photo tourism, usually with low‐speed traffic. The main paved road in the study area is surrounded by the highest development in the area and is generally avoided by African wild dogs. Roads in all four regions are predominantly seasonal tracks that do not allow high speed, reducing the risk for African wild dogs. There were no observed disease‐related African wild dog deaths in the study area. No individuals were recorded losing condition due to disease or showing symptoms of rabies, distemper, or anthrax, which can cause significant mortality in African wild dogs. It was not possible to confirm the cause of death for most individuals (particularly for young pups) but our ability to detect disease‐caused deaths, if they occurred, should have been equal across the four regions. The North, Nsefu, and Lower Lupande regions are similarly exposed to communities with domestic dogs and would likely be exposed to similar disease risks (Prager et al., 2012; Woodroffe et al., 2012). There were no known incidents of African wild dogs being poisoned or shot during the study period.

The average pack size in the LVE (5.27 adults and 7.60 adults and yearlings) was relatively small, in comparison to other high‐density African wild dog populations (Creel et al., 2004; Goodheart et al., 2021). For African wild dogs, pack size is typically related to prey size (Creel & Creel, 2002; Mills & Gorman, 1997) although small prey at high densities (i.e., 138.7 dikdiks/km^2^) can sometimes support large packs (Woodroffe, Lindsey, et al., 2007), and the relatively small packs in the LVE are consistent with their diet of relatively small antelopes (see Methods: impala, puku, and bushbuck are the most common prey). Unlike African wild dogs in some other ecosystems (i.e., Kruger and Selous) (Creel & Creel, 2002; Mills & Gorman, 1997), large packs did not switch to larger prey (particularly wildebeest), probably because wildebeest are not common in the LVE (East, 1989; Estes, 2014; Northern, 1934). The Cookson's wildebeest (*Connochaetes taurinus cookson*i) in the LVE are estimated from 1000 to 6000 individuals compared to Selous Game Reserve in Tanzania where Nyassa wildebeest (*Connochaetes taurinus johnstoni*) abundance is an order of magnitude greater at ~50,000 to 75,000 and wildebeest are common prey for African wild dogs (Creel & Creel, 2002); or to Liuwa Plain National Park in western Zambia where blue wildebeest (*Connochaetes taurinus taurinus*) abundance has been estimated between 23,500 to 35,000 and densities of 6.2 to 60.8 individuals/km^2^ and they are common prey for African wild dogs (Dröge et al., 2017; East, 1989; Estes, 2014; Northern, 1934). African wild dogs in the LVE may also avoid larger prey items to minimize risk of kleptoparasitism or intraguild predation from dominant competitors.

The low survival and reproductive success of African wild dogs in Lower Lupande suggest that the region is a demographic sink. If prey‐depleted areas like Lower Lupande are sinks, it is likely that regions with higher prey densities, particularly Nsefu, are sources. Further investigation is needed to directly test for source–sink dynamics driven by prey depletion and to identify the threshold in prey density that can shift a source habitat into a sink. The detrimental effects of prey depletion on African wild dog fitness despite lower lion densities indicate that some protected areas (and likely many unprotected areas) are approaching a threshold that is unfavorable for African wild dog populations.

Endangered species like the African wild dog serve as indicators of the threats driving the broader extinction crisis (Ceballos et al., 2020). The degradation of protected areas (PAs) is creating a precarious situation, given that the world's current PA network is projected to protect only half of all mammals from anthropogenic extinction (Williams et al., 2022). Continued habitat degradation, such as anthropogenic prey depletion, is likely to cause an increased risk of extinction and loss of ecosystem function. The number and size of PAs are key metrics used in conservation planning (i.e., African wild dog) and the erosion of those PAs' effectiveness will alter conservation priorities (Kuiper et al., 2018). Degraded PAs (i.e., GMAs IUCN IV) could be prime areas for investment to mitigate biodiversity loss and critical for guiding conservation action plans (Lindsey et al., 2014). African PAs near areas of high human activity require more resources and management to mitigate negative anthropogenic effects. Novel, inclusive, globally linked, and community‐centered approaches will be necessary to better protect PAs and adjacent areas (Berkes, 2017; Lindsey et al., 2020). While anthropogenic pressure is often correlated with negative impacts, there is potential to integrate communities into conservation efforts with the use of local indigenous knowledge, cultural heritage, and direct benefits to the community that aligns with conservation goals (Gavin et al., 2018; Magness et al., 2022; Mavhura & Mushure, 2019). Investment from across the globe will be required to maintain, strengthen, and grow PAs to protect ecosystems, species, and human communities and to meet interlinked sustainable development goals (Krause & Tilker, 2022).

## 4 MATERIALS AND METHODS

### 4.1 Study area and regions

The Luangwa Valley Ecosystem (LVE) lies in eastern Zambia (S 12.909338, E 31.918769) and is comprised of four National Parks (South Luangwa, Luambe, Lukusuzi, and North Luangwa) and seven Game Management Areas (GMAs). A network of GMAs, Game Reserves, and Forest Reserves connects the LVE to Transfrontier Conservation Areas (TFCAs) shared with Malawi, Zimbabwe, and Mozambique (Andersson et al., 2017). The LVE is surrounded by escarpments of the Luangwa Rift and hills with elevations ranging from 1000 to 1500 m above sea level, while the basin ranges from 500 to 800 m (Banks et al., 1995). The ecosystem contains a mix of mopane (*Colophospermum mopane*) woodlands, miombo (*Brachystegia* spp.) woodlands, riparian woodlands, scrublands, and open grasslands (Astle et al., 1969; Rosenblatt et al., 2014). The LVE has three main seasons: dry‐cool (May–July), dry‐hot (August–October), and rainy (November–April). The average annual rainfall in the LVE ranges from 700 to 900 mm, with majority of the rain falling during the rainy season (Astle, 1999; Astle et al., 1969; Dewald et al., 2023; Shrader et al., 2010).

Our study focused on the Southern Luangwa Valley, an area of 6938 km^2^ centered on the Luangwa River and includes the east of South Luangwa National Park (SLNP), the west of Luambe National Park (LNP), and portions of three GMAs (Munyamadzi, Lumimba, and Lupande). The Luangwa River forms the backbone of the LVE as it flows south/southwest to join the Zambezi River. Wildlife is distributed throughout the study area, but wildlife density is highest along the Luangwa River, particularly in the dry season (Rosenblatt et al., 2019). The Luangwa River is the eastern boundary for most of SLNP and the western boundary for LNP. The Muphamadzi River is the second‐largest perennial river in the study area and forms part of the northern boundary of SLNP. The only all‐year tarred road bisects Lupande GMA (creating Upper and Lower Lupande, respectively) and connects SLNP to the town of Mfuwe with a small international airport, and then to the district capital (Jumbe) and provincial capital (Chipata). SLNP is the second largest National Park in Zambia (8704 km^2^) and has a prominent role in Zambia's tourism economy (Mvula, 2001). The town of Mfuwe (62,000 people within 30 km of SLNP) benefits from the tourism economy, although the majority of the population is engaged in agriculture, primarily through subsistence farming (Chidakel et al., 2021).

The GMAs fall under IUCN Category VI, which allows resource harvesting (including professional hunting concessions) and communities to reside within the GMA. The Department of National Parks and Wildlife manages SLNP (IUCN category II) under a high level of protection with no permitted resource harvesting or human settlements. The GMAs are exposed to more wire‐snare poaching, human–wildlife conflict, and habitat conversion than SLNP (Watson et al., 2013, 2015).

Thus, the Luangwa River is a semi‐permeable boundary with very similar natural habitat (i.e., vegetation, access to water, and rainfall) on both sides, but different levels of protection and management mandates. For African wild dogs, these differences in protection create strong differences in the densities of both prey and lions. To test how these differences in prey and dominant competitors affect the demography and density of African wild dogs, we identified four distinct regions within our study area:

1. *Main Game*: a 2209 km^2^ region of SLNP that comprises the main photographic tourism area and is west of the Luangwa River and has the highest relative protection level.
2. *Nsefu*: a 1318 km^2^ region in the Nsefu sector of SLNP, portions of the Upper Lupande GMA, and the southern portion of Lumimba GMA (south of Lukuzye River), with intermediate protection.
3. *North*: an 1801 km^2^ region in the western portion of Luambe NP, the northeastern portion of SLNP along the Muphamadzi River, the northern portion of Lumimba GMA (north of Lukuzye River), and portions of the Munyamadzi GMA, with intermediate levels of protection. This region includes areas on both the east and west sides of the Luangwa River with similar mosaics of protection levels. This region had limited data collection relative to the other three regions.
4. *Lower Lupande*: a 1610 km^2^ region in the Lower Lupande GMA (south of the paved road) and the Lusangazi sector of SLNP, which lies to the east of the Luangwa River. This region has a relatively low protection level.

Arranging these regions from highest to lowest level of protection, the sequence is

Main Game>Nsefu≥North>Lower Lupande.

The effective level of protection in the North region is not as well described as the other three regions (Becker et al., 2013; Rosenblatt et al., 2016, 2019; Watson et al., 2013), but it is certainly lower than Main Game and higher than Lower Lupande. The differences in anthropogenic pressure and protection levels between these regions produce previously described variations between the regions in the risk of injury or death by wire snares, prey density, and lion density (Mweetwa et al., 2018; Rosenblatt et al., 2014, 2016, 2019; Watson et al., 2013, 2015).

The Main Game region is solely within SLNP (IUCN category II) with photo‐tourism and management activities as the primary human footprint. The Nsefu region is a mosaic of NP and GMAs (IUCN category VI) with photo‐tourism in the NP, trophy hunting in the GMA, and high human traffic moving between settlements on a dirt track by foot, bicycle, and vehicle. The North region is also a mosaic of NPs and GMAs, but it is remote and inaccessible, particularly during the rainy season. It has historically received less investment in management and photo‐tourism but has no major track and overall low‐throughput traffic. The Lower Lupande region is primarily located in the GMA with photo‐tourism, trophy hunting, and heavy human use, including considerable illegal wire snaring (Watson et al., 2013).

These differences between regions create a gradient in the intensity of wire‐snare poaching, as follows:

Main Game<North≤Nsefu≤Lower Lupande.

We determined prey density twice annually since 2013, by fitting distance sampling models to observations from a fixed grid of ground transects. As described in detail by Rosenblatt et al. (2019), these transects were surveyed twice each dry season (May to Oct), in the Main Game, Nsefu, and Lower Lupande regions (Rosenblatt et al., 2019). Distance sampling using the same methods was conducted in the North region from 2018 to 2020. The diet of African wild dogs in LVE based on proportion of observed kills (*N* = 322) was impala (*Aepyceros melampus*) at 64.29%, followed by puku (*Kobus vardonii*) at 20.81%, and bushbuck (*Tragelaphus scriptus*) at 9.63%. All other species contributed less than 1% except scrub hare (*Lepus saxatilis*) at 1.63% and waterbuck (*Kobus ellipsiprymnus*) at 1.31%. Rosenblatt et al. (2019) reported impala densities of 37.33 (95% CI: 32.44–42.56) individuals/km^2^ in Nsefu, 31.73 (95% CI: 26.07–37.60) in Main Game, and 8.47 (95% CI: 6.22–10.61) in Lower Lupande (Rosenblatt et al., 2019). Puku densities followed a similar pattern with a mean of 24.43 individuals/km^2^ (95% CI: 16.90–32.16) in Nsefu, 5.94 (95% CI: 4.73–7.20) in Main Game, and 2.69 (95% CI: 1.72–3.66) in Lower Lupande (Rosenblatt et al., 2019). The North region is estimated to have a median of 13.76 (95% CI: 10.46–17.96) impala/km^2^ and 4.94 (95% CI: 3.68–6.47) puku/km^2^ (unpublished Zambian Carnivore Programme report). The regional prey density estimates are summarized in Table 2.

**TABLE 2:** Summary of regional prey density (individuals/ km^2^) estimates in the Luangwa Valley Ecosystem.

	Region	Impala	Puku	Impala & Puku
	Nsefu	37.33 (32.44–42.56)	24.43 (16.90–32.16)	61.76 (49.33–74.72)
	Main Game	31.73 (26.07–37.60)	5.94 (4.73–7.20)	37.67 (30.80–44.80)
	North	13.76^a^ (10.46–17.96)	4.94^a^ (3.68–6.47)	18.70^a^ (14.14–24.43)
	Lower Lupande	8.47 (6.22–10.61)	2.69 (1.72–3.66)	11.16 (7.94–14.26)

*Note*: Mean estimates and 95% confidence intervals for impala, puku, and a combined measure of both impala and puku. The color shades of orange, black, blue, and red match the reference colors for regions in other figures.

^a^ Refers to the median.

Thus, the differences between regions create a gradient in the density of prey, as follows:

Nsefu≥Main Game>North>Lower Lupande.

Lions have been intensively monitored in Nsefu, Lower Lupande, and the Main Game region since 2008 (Mweetwa et al., 2018; Rosenblatt et al., 2014), and since 2018, in the North, using the same methods described here for African wild dogs. We ranked the regions by lion density using the number of known adult and subadult lions in each region (from 2018 to 2020) divided by the area that they occupied, which we determined from merged 95% dynamic Brownian bridge utilization distribution for those individuals. Estimates of total population size from capture–mark–recapture analysis have been similar to the number of known lions in our prior research on this population (Mweetwa et al., 2018; Rosenblatt et al., 2014), but the data were not sufficient to fit such models separately for each region. The estimated mean lion density was 10.46, adult and subadult lions/100 km^2^ for Main Game, 9.25 for Nsefu, and 4.02 for Lower Lupande. The North region was less intensively sampled but held an estimated 3.25 known adult and subadult lions/100 km^2^. The estimate for the North region is not directly comparable to other regions as lion monitoring effort was limited, which led to fewer intensively monitored prides and excluded several undermonitored prides in the overlapping monitored area. The excluded prides included uniquely identified adult and subadult lions for which we had only limited spatial data. In unpublished data from acoustic surveys, the North region was estimated to have 86.6% of the lion density in the Main Game region (which would yield 9.07 adult and subadult lions/100 km^2^) (unpublished Zambian Carnivore Programme report). The 2023 estimate for the North region was 8.11 known adult and subadult lions/100 km^2^, and our monitoring in 2023 was more comparable to other regions in 2018–2020. We believe that the 2023 estimate for the North best represents the region's lion density and used this value to rank the region.

The regions arranged from highest to lowest lion density are as follows:

Main Game>Nsefu>North>Lower Lupande.

### 4.2 Monitoring

In partnership with the Zambia Department of National Parks and Wildlife (DNPW), we began monitoring African wild dogs in 2008 by radiocollaring 1–2 members of each pack, developing a photographic identification database for all individuals, and recording survival and reproduction by frequent direct observation. Here, we analyzed data for the period from 2014 to 2020. For these years, approximately 1000 person‐days/year were committed to direct observations that generated 9685 sightings of 491 individually identified African wild dogs in 40 packs or single‐sex groups of dispersers. Our methods of detection, monitoring, and identification of individual African wild dogs have been previously described (Creel & Creel, 2002; Goodheart et al., 2021). African wild dogs have highly unique coat patterns of black, tan, and white that allow reliable identification using photographs, and sex is easily determined for all age classes. Of the 491 individuals with data analyzed here, 25 juveniles and three adults died or dispersed before having their sex identified. These individuals were assigned unknown sex (*n* = 28). For individuals first identified as adults, age was estimated based on body size, tooth wear, and pelage, but because prior research shows that adult African wild dogs have relatively constant survival (Creel et al., 2004), our analysis binned individuals into three age categories: pups (0–0.99 years old), yearlings (1–1.99 years old), and adults (≥2 years old). Error in assignment to these categories is unlikely.

We used VHF, GPS Store‐On‐Board, and GPS Iridium satellite collars (Telonics Inc., Mesa, Arizona, USA) to relocate African wild dogs, and 28 of the 40 groups had at least one collared group member. In periods when a group did not carry a collar, it was monitored through a combination of opportunistic sightings and photographs provided by citizen science through guides, tourists, tourist operators, professional hunters, program partners, and DNPW staff. The four regions that we identified for analysis exclude areas in which dogs were present but not monitored adequately. All GPS collars collected a minimum of two locations/day. The highly cohesive nature of African wild dog packs allows reliable monitoring of all individuals in packs with a radiocollar. At every sighting, the location (GPS coordinates), date, time, and identified individuals were recorded. If an individual identification was uncertain, photos were taken with a digital camera to allow later verification.

Radiocollars were deployed by immobilization via intramuscular injection of 20 mg Zoletil and 1.2 mg Medetomidine mixture, delivered by dart using a DanInject air rifle. All immobilizations were performed by a Zambian‐registered veterinarian in partnership with the Zambian DNPW, and with MSU (Montana State University) IACUC approval. We used an intramuscular injection of Atipamezole to reverse Medetomidine at 45–60 min as the effects of Zoletil waned, typically producing recovery that allowed the dog to walk within 20 min. Individuals were routinely checked for injuries with a special emphasis on snare wires or injuries on the neck, legs, or torso. If an individual was snared, the same immobilization protocol was used to remove the snare wire and treat the wound.

### 4.3 Population density

#### 4.3.1 Population size

We estimated abundance ($\hat{N}$) using Bayesian methods to fit a closed mark–recapture (CMR) model to African wild dog detections in each year from 2014 to 2020 (Otis et al., 1978). The CMR model allowed for individual variation in detection probability (*p*) by including a random effect with a Gaussian distribution on a logit scale (Kéry & Schaub, 2012). For each year, we fit this model to detections that were binned into seven monthly occasions from May to November. We excluded data from December to April because the frequency of detection decreased considerably during the peak of the wet season, and to better meet the model's assumptions (also see tests for goodness of fit below).

Because most prior estimates of African wild dog density exclude pups less than 1 year old, we included only adult and yearling African wild dogs in the annual abundance estimates to allow direct comparison (Creel et al., 2004; Woodroffe, 2011). All individuals that were known to have died or dispersed outside of the annual demographic monitoring area were excluded from the CMR model. To address the model's closure assumption, only individuals known to be alive before and after the 7‐month window were included in the data to which the closed CMR model was fit. After fitting the model, any individuals that were removed (average of 17% of known individuals per year) were added back to the abundance estimate for that year. We fit the model with uninformative prior distributions using three Markov chains, with 25,000 iterations and 5000‐step burn‐in using the R package R2Jags (Yu & Yajima, 2012), using the data augmentation method of Royle et al. (2007) (Royle et al., 2007). We confirmed the model's fit using trace plots, by confirming that R‐hat values were close to 1 for all parameters, by posterior predictive checks showing that capture histories simulated under the model matched the original data well, and with a Q‐Q plot confirming that a logit‐normal random effect provided a good fit to individual variation in detectability. The estimate of annual African wild dog abundance ($\hat{N}$) was divided by the annual demographic monitoring area ($\hat{A}$, described below) to estimate density ($\hat{D}$).

#### 4.3.2 Area utilized

The exact demographic monitoring area varied between years, as packs formed and dissolved, home ranges shifted, and our monitoring effort responded. Most of the area within the four regions of the study site was monitored in all the years between 2014 and 2020, but the exact boundaries varied sufficiently to affect estimates of density. Thus, we estimated the annual home range boundary for each group included in that year's density estimate to allow for changes in the demographic monitoring area. The annual home ranges of all groups were merged (with all internal boundaries dissolved) to define the annual demographic monitoring area. We used two methods (kernel utilization distribution and dynamic Brownian bridge movement model) to determine annual home range boundaries. We fit kernel utilization distributions (KUDs) to allow backward comparability with prior estimates of African wild dog density in other ecosystems (Worton, 1989). The dynamic Brownian bridge movement model (dBBMM) better accounts for spatial and temporal autocorrelation in locations from GPS collars when estimating the utilization distribution of the collared animal (Kranstauber et al., 2012). We view estimates based on dBBMMs as a better description of density, but (because dBBMMs predictably exclude unused areas both inside and at the edges of a range, when compared to KUDs fit to the same data) dBBMMs estimate smaller ranges than KUDs, so we report both estimates to allow comparison to both past and future studies.

We determined the annual KUD for each group by using the *adehabitatHR* package in R (Calenge, 2006) to determine the 95th percentile isopleth of a KUD fit to all locations from GPS collars, VHF tracking, and opportunistic sightings. We used the *move* package in R (Kranstauber et al., 2018) to identify the 95th percentile isopleth of a dynamic Brownian bridge movement model (dBBMM) from GPS locations. Following methods from Goodheart et al. (2022) for data from the same radiocollars on African wild dogs, the window size was set to 15 fixes, margin size to 3 fixes, and location error to 1 m (Goodheart et al., 2022). We estimated density ($\hat{D}$) using the dBBMM‐based estimate of area in only 4 years (2016, 2018, 2019, and 2020) for which we had GPS collar data for all groups in all four regions of the focal study area. We averaged these estimates across the 4 years to provide a single point of comparison to other ecosystems. We estimated density ($\hat{D}$) using the KUD‐based estimate of area for each of the 7 years (2014–2020). We averaged these estimates across 7 years to provide a single point of comparison to other ecosystems.

### 4.4 Survival

#### 4.4.1 Age‐ and sex‐specific annual apparent survival (ϕ)

To estimate age‐ and sex‐specific annual survival rates, we fit a Cormack–Jolly–Seber (CJS) model to monthly detection histories for 463 known‐sex individuals from 2014 to 2020, using Bayesian methods (Kéry & Schaub, 2012; Royle, 2008; Seber, 1965). Following the methods of Kéry and Schaub (2012), the CJS model estimated annual apparent survival (ϕ) after correction for the probability of detection (*p*). As in the model of abundance (above), we allowed *p* to vary among individuals by fitting a random effect with a Gaussian distribution on the logit scale. We binned detections into nine monthly occasions (April to December) for every year, for a total of 63 occasions across the 7 years (2014–2020), and 4082 unique monthly detections. Three months (Jan–March), the peak of the rainy season, were not included due to low rates of detection. We estimated apparent survival rates (ϕ) for each sex and three biologically meaningful age classes: pups (<1 year old), yearlings (1 year old), and adults (≥2 years old). Each individual that crossed an age class boundary was shifted on the 1st of June each year (the onset of the birth season). We fit the CJS model with three Markov chains of 4500 steps after a 500‐step burn‐in and with uninformative uniform prior distributions for both *p* and ϕ. We confirmed the model's fit using trace plots, by confirming that R‐hat values were close to 1 for all parameters, by posterior predictive checks showing that capture histories simulated under the model matched the original data well, and with a Q‐Q plot confirming that a Gaussian distribution logit scale provided a good fit to individual variation in detectability. We also tested whether radiocollaring had an effect on adult survival rates using a Bayesian Cormack–Jolly–Seber model that controlled for individual variation in the probability of detection.

#### 4.4.2 Region‐specific annual apparent survival (ϕ)

Our data were not sufficient to estimate age‐ and sex‐specific survival rates (ϕ) for each of the four regions, so to test for variation among the four regions, we fit a second CJS model, again with individual random effects on detection probability (*p*, with a Gaussian distribution on the logit scale), and using the same time bins as the prior analysis. Separately, we examined the distribution of individuals among age classes in each of the regions, to confirm that differences in population structure could not explain differences between regions in estimated survival rates (see Section 2). Each of the 463 African wild dogs was assigned to one of the four regions for each of the 63 sampling occasions in which it was detected. Overall, most individuals had high fidelity to a single region so short‐term shifts to other regions were rare, but dispersing animals often settled in a new region. Dispersing animals that were not detected in a month between the last sighting prior to dispersal and the first sighting after dispersal were assigned to the region in which they settled. We fit the CJS model with three Markov chains of 4500 steps after a 500‐step burn‐in, with uninformative prior distributions for both *p* and ϕ. We confirmed the model's fit using trace plots, by confirming that R‐hat values were close to 1 for all parameters, by posterior predictive checks showing that capture histories simulated under the model matched the original data well, and with a Q‐Q plot confirming that a random effect with a logit‐normal distribution provided a good fit to individual variation in detectability. Goodheart et al. (2021) estimated annual apparent survival (ϕ) for African wild dogs in the Greater Kafue Ecosystem (GKE), where both lion and prey densities are very low, using the same model and methods (Goodheart et al., 2021). The GKE estimate was used in conjunction with the four region‐specific LVE estimates to compare the effects of lion and prey density on African wild dog survival.

### 4.5 Litter size and recruitment

We tested for differences between regions in litter size at first count, the number of pups raised to 1 year, and the recruitment ratio (proportion of pups raised to 1 year). We directly observed all packs frequently, using VHF telemetry and downloaded satellite/GPS collar locations. Reproduction is highly seasonal in African wild dogs, and because they produce the heaviest litters relative to female body size of all carnivores, pregnancy is easily detected (Creel & Creel, 1991, 2002). In a successful pregnancy, the signs become increasingly apparent until parturition. Lactation is also easily detected. Pregnant females also engage in conspicuous denning behavior, exploring and excavating burrows, and African wild dogs rarely rest in the same location for two consecutive days except when they are denning (Creel & Creel, 2002; Malcolm & Marten, 1982). During the denning period, all pack members return to the den site after most hunts. During hunts in the denning period, the breeding female (and sometimes others) usually remains at the den to guard the pups. Using all these criteria, we located dens for all packs and monitored the number of pups that emerged and survived to 1 year.

Because African wild dogs are cooperative breeders and a single female reproduces in most cases (with exceptions, see below), we report the number of pups produced and raised by each pack in each year. We defined a pack as a group with at least one unrelated adult of each sex that resided within the study area during the year of interest. If a pack did not produce any offspring during the year (or failed to raise any pups) they were assigned a litter size/recruitment of zero. The offspring of subordinate females that produced a litter were added as additional data points (Pack‐Year‐Beta) for both litter size and recruitment. This occurred twice, once in the North region and once in the Main Game region.

Because pups remain underground in the first weeks of life, litter sizes were counted at 45 days (±15 days) from the estimated date of birth. During the month postpartum, dens were located and routes were planned to access and approach each den. Den visits began 1 month after the onset of denning, and pups were counted, photographed, identified, and had their sex determined. Den visits were conducted from a vehicle and sought to minimize disturbance in time windows after morning hunts or before evening hunts, as they provided the best opportunities to observe pups outside the den. We excluded pack‐years with litters that were not counted in this window from our analysis. Observations of litter size include some measurement error because pups are born underground, and not counted until they emerge approximately 1 month later. To minimize disturbance, we avoided dens for the first month but then prioritized making an accurate count of the pups and keeping the time at which litter size was measured consistent (Moreover, our results showed that differences between regions were consistent for all three measures of reproduction).

Recruitment was measured as the number of pups that survived the denning season of the following year (June 15th). We recorded the number of pups recruited even if the initial litter size was not known. The recruitment ratio was measured as the number of pups that survived to the next year divided by the initial litter size when both were known.

We tested for variation among regions in litter size, recruitment, and recruitment ratios using data from 2008 to 2021, accounting for pack size, which has strong effects on reproductive success in African wild dogs (Courchamp & Macdonald, 2001; Creel et al., 2004; Creel & Creel, 2015; Gusset & Macdonald, 2010; Malcolm & Marten, 1982; McNutt & Silk, 2008). Data restricted to the 2014–2020 interval show the same trends as the 2008–2021 data, which provided a larger sample (*N* = 71 pack‐years for litter size, 80 pack‐years for pups recruited to 1 year, and 49 for recruitment ratio). Pack size was measured as the total number of adults (≥2 years old) in the group when the den was established (typically mid‐June). Any pack‐year for which we did not record pack size at the onset of denning was excluded from the analysis.

#### 4.5.1 Litter size model

We used Bayesian methods to fit a generalized linear model (GLM) with a Poisson distribution to data on litter size for 71 pack‐years from 2008 to 2020. The GLM was fit with three Markov chains of 4000 steps after a 1000‐step burn‐in, with uninformative uniform prior distributions for intercept, differences in region, and pack size. We confirmed that no inferences were altered by adding a hurdle for zero inflation, or by fitting a model with a negative binomial distribution and that a model with linear and quadratic effects of group size fit better than a model with each of these effects in isolation.

#### 4.5.2 Raw recruitment to 1‐year model

We used Bayesian methods to fit a generalized linear model (GLM) with a Poisson distribution to data on recruitment for 80 pack‐years from 2008 to 2020, with effects of region and both a linear and a quadratic effect of pack size. The GLM was fit with three Markov chains of 4000 steps after a 1000‐step burn‐in, with uninformative uniform prior distributions for intercept, differences in region, and pack size.

#### 4.5.3 Recruitment ratio model

We used Bayesian methods to fit a beta regression to data on the recruitment ratio of 49 pack‐years from 2008 to 2020. Using the R package Brms (Bürkner, 2017), we modeled the recruitment ratio as a quadratic function of pack size, with variation among regions for the mean, mu (*μ*), and variation among regions for precision, phi (ϕ). The dependent variable (recruitment ratio) was transformed to keep the values greater than 0 and less than 1 (but not equal to either) as a beta regression, following Smithson & Verkuilen, 2006 (Smithson & Verkuilen, 2006), where y is the dependent variable and *n* is the sample size:

$$\frac{\left(y*\left(n-1\right)+0.5\right)}{n}$$

The beta regression was fit with three Markov chains of 2000 steps after a 1000‐step burn‐in and with uninformative uniform prior distributions for the mean, mu (μ), and precision, phi (ϕ).

## AUTHOR CONTRIBUTIONS

**Johnathan Reyes de Merkle:** Conceptualization (equal); data curation (lead); formal analysis (lead); investigation (equal); methodology (supporting); software (equal); visualization (lead); writing – original draft (lead); writing – review and editing (lead). **Scott Creel:** Conceptualization (equal); formal analysis (supporting); funding acquisition (equal); methodology (equal); project administration (supporting); software (equal); supervision (equal); writing – original draft (supporting); writing – review and editing (supporting). **Matthew S. Becker:** Conceptualization (equal); funding acquisition (equal); investigation (equal); methodology (equal); project administration (lead); supervision (equal); writing – review and editing (supporting). **Ben Goodheart:** Investigation (supporting); methodology (supporting); software (supporting); writing – review and editing (supporting). **Thandiwe Mweetwa:** Investigation (supporting); project administration (supporting). **Henry Mwape:** Investigation (supporting). **Egil Dröge:** Data curation (supporting); investigation (supporting); methodology (supporting). **Twakundine Simpamba:** Investigation (supporting); project administration (supporting); resources (supporting).

## CONFLICT OF INTEREST STATEMENT

The authors declare no conflict of interest.

## Supporting information


Data S1.

## Data Availability

The data that support the findings of this study are openly available in Dryad at https://doi.org/10.5061/dryad.qbzkh18q0.
