# Supplementary material for: Long‐term data reveal fitness costs of anthropogenic prey depletion for a subordinate competitor, the African wild dog (Lycaon pictus)
Source: Ecol Evol. 2024 Jun 25;14(6):e11402. doi: 10.1002/ece3.11402 (PMC11199200; doi:10.1002/ece3.11402)
Supplement: Supplementary file 1 — Data S1. [file ECE3-14-e11402-s001.docx]

**Supporting Information for**

**Long-term data reveal fitness costs of anthropogenic prey depletion for a subordinate competitor, the African wild dog (*Lycaon pictus*)**

Johnathan Reyes de Merkle ^a,b^, Scott Creel ^a,b,c^, Matthew S. Becker ^a,b^, Ben Goodheart ^a,b^, Thandiwe Mweetwa ^a^, Henry Mwape ^a^, Egil Dröge ^a,b,d^, Twakundine Simpamba ^e^

^a^ Zambian Carnivore Programme, PO Box 80, Mfuwe, Eastern Province, Zambia

^b^ Department of Ecology, Montana State University, Bozeman, MT 59717, USA

^c^ Institutionen för Vilt, Fisk och Miljö, Sveriges lantbruksuniversitet, Umeå, Sweden

^d^ Wildlife Conservation Research Unit, Oxford University, Oxford OX1 2PS, England

^e^ Department of National Parks and Wildlife, South Luangwa Area Management Unit, Mfuwe, Eastern Province, Zambia

**Corresponding Author:** Johnathan Reyes de Merkle

**Email:**  JohnathanReyesdeMerkle@gmail.com

**This file includes:**

Figure S1

Figure S2

Table S1

Table S2

Table S3

Additional comments

**Fig. S1.**

**
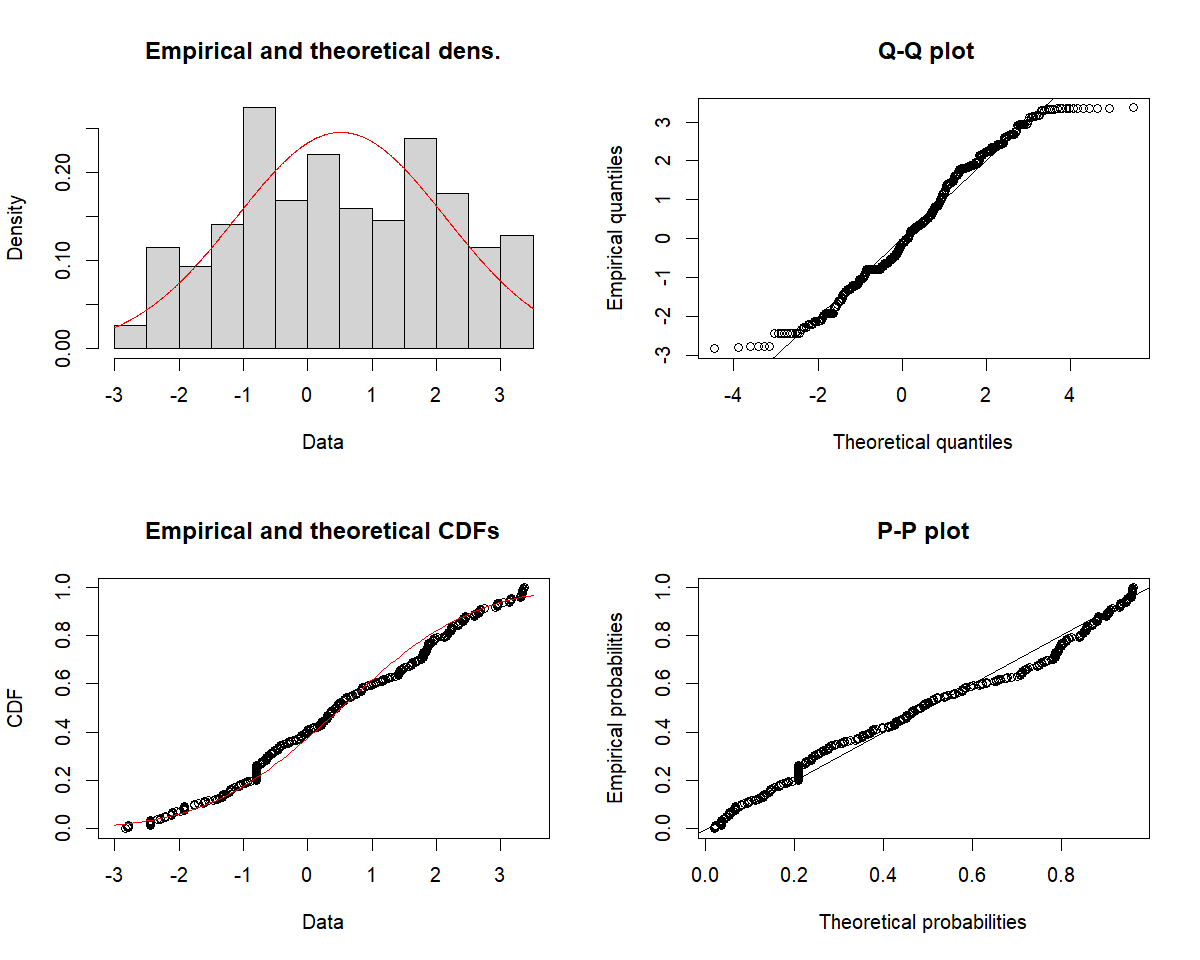
**

Goodness of Fit (GOF) plot for Cormack Jolly-Seber model based on monthly detection histories for 463 known-sex individuals from 2014 to 2020.

Additional summary statistics of samples:

Table S1

| **Sex** | **Count** |
| --- | --- |
| Female | 220 |
| Male | 243 |
| Unknown | 28 |
| Total | 491 |

Summary of sex distribution of monitored African wild dogs (2014-2020).

Table S2

| **Percentage of monthly detections by age class** | |  |
| --- | --- | --- |
|  |  |  |
| ***Age Class*** | ***Percentage*** |  |
| Pup (<1 year) | 25.91% |  |
| Yearling (1 to <2 years) | 20.75% |  |
| Adult (>2 years) | 54.34% |  |

Summary of the percentage of monthly detections by age class (2014-2020).

Table S3

| **Percentage of monthly detections by year** | |  |
| --- | --- | --- |
|  |  |  |
| ***Year*** | ***Percentage*** |  |
| 2020 | 16.36% |  |
| 2019 | 12.96% |  |
| 2018 | 16.43% |  |
| 2017 | 17.14% |  |
| 2016 | 13.13% |  |
| 2015 | 14.43% |  |
| 2014 | 9.55% |  |

Summary of the percentage of monthly detections by year (2014-2020).

Reproduction :

Litter size:

The total number of pups counted was 438 from 2008-2021.

Recruitment to one year:

A total of 231 pups recruited from 2008-2021.

Fig S2


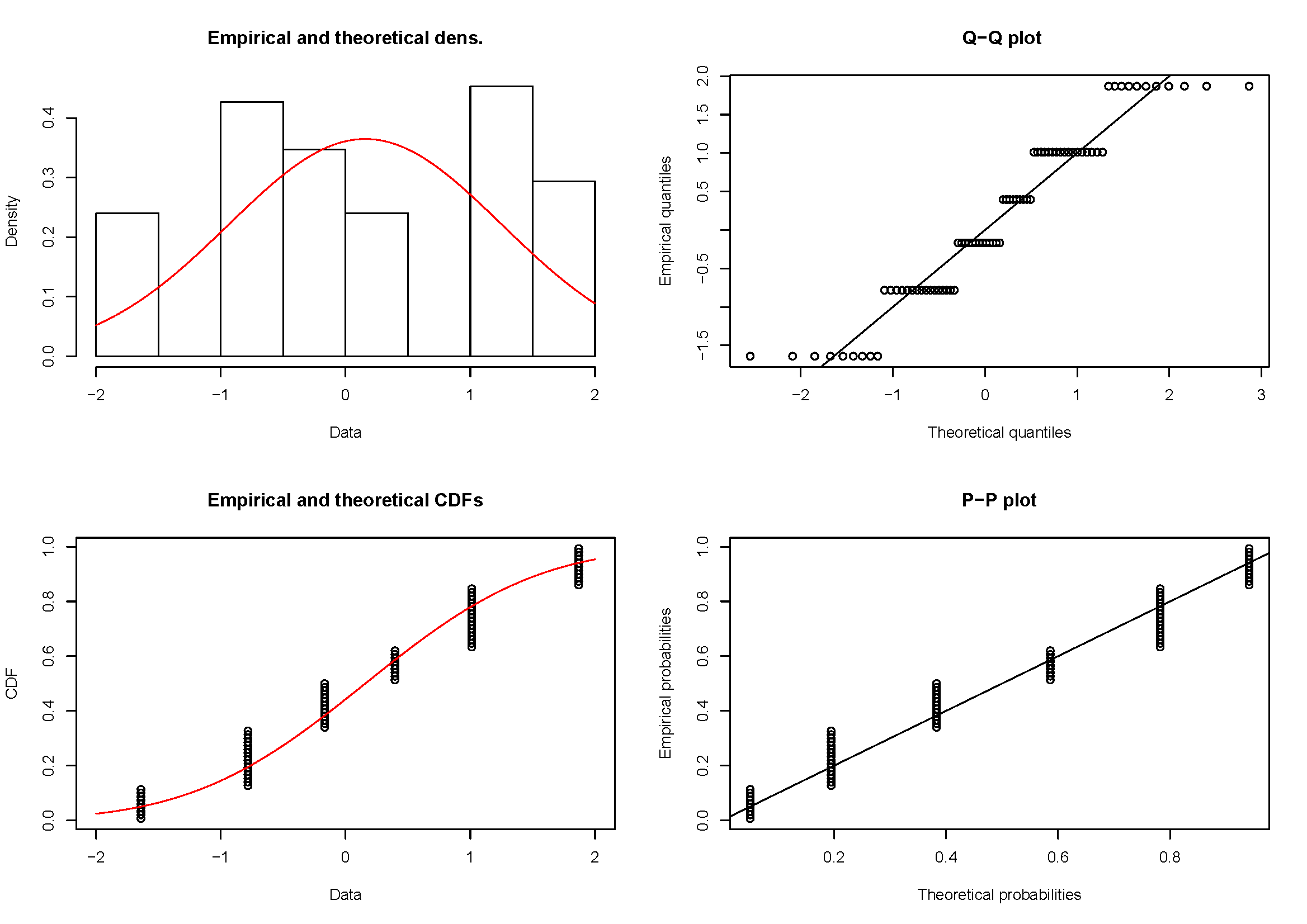


Goodness of Fit (GOF) plot for the closed mark-recapture (CMR) based on seven monthly (May-October) detection histories for 81 individuals in 2019.
